# Using a process improvement approach to identifying barriers to research in a CTSA hub environment

**DOI:** 10.1017/cts.2020.522

**Published:** 2020-08-19

**Authors:** Douglas Grabenstetter, Ramez Rashid, Jeff Whittle

**Affiliations:** 1 Mechanical Engineering Department, Industrial Engineering Program, Milwaukee School of Engineering University, Milwaukee, WI, USA; 2 Clinical Translational Science Institute, Medical College of Wisconsin, Milwaukee, WI, USA; 3 Department of Medicine, Division of General Internal Medicine, Medical College of Wisconsin, Milwaukee, WI, USA; 4 Primary Care Division, Clement J. Zablocki VA Medical Center, Milwaukee, WI, USA

**Keywords:** Barriers, translational research, continuous improvement, perceptions, policy, qualitative research, quantitative research, Six Sigma, multicriteria decision-making

## Abstract

For the past 4 years, as part of the National Institutes of Health (NIH) Clinical and Translational Science Award (CTSA) grant award number UL1TR001436, the Clinical Translational Science Institute of Southeast Wisconsin (CTSI) has used process engineering approaches to identify and understand barriers that local researchers and other stakeholders face when engaging in clinical and translational science. We describe these approaches and present preliminary results. We identified barriers from published and unpublished work at other CTSA hubs, supplemented by surveys and semi-structured interviews of CTSI faculty. We then used a multifaceted approach to organize, visualize, and analyze the barriers. We have identified 27 barriers to date. We ranked their priority for CTSI to address based on the barrier’s impact, the feasibility of intervention, and whether addressing the barrier aligned with CTSI’s institutional role. This approach provides a systematic framework to scope and address the “barriers to research problem” at CTSI institutions.

## Introduction

While the past two decades have experienced significant advancements in science and medicine, various sources suggest that the average time for scientific findings to be translated into improvements in public health exceeds 10 years [1–3]. In part to address this delay, the National Institutes of Health’s National Center for Advancing Translational Sciences (NCATS) has funded over 55 Clinical and Translational Science Award (CTSA) program hubs. CTSA hubs have been tasked to work individually and as a group to speed the translation of research discoveries into improved health and healthcare. This task requires that hub leaders identify key barriers, prioritize where efforts are most likely to yield benefits and appropriately allocate resources. This complex mission is made more difficult by the need to collaborate across organizational entities within a hub and within the CTSA network. Further, within each academic medical center, there are many processes that impact research projects at a macro level, but do not belong to, and often cannot be addressed by, anyone in the research team.

To address similar challenges, industries from automobile [4] and pharmaceutical [5–8] manufacturing to healthcare provision [9–11] have used Lean and Six Sigma process (quality*)* improvement methods. Further, research has pointed out that CTSA processes lend themselves to Lean Six Sigma (LSS) approaches [12]. While a limited number of published works have examined the use of these approaches in the CTSA domain [12–14], we found no examples of the use of LSS tools as part of an *integrated methodology* to visualize, identify, and prioritize opportunities to address barriers to performing research, one of the key tasks for CTSA hubs. In our paper, we present the approach we have used over the last 3 years at the Clinical Translational Science Institute of Southeast Wisconsin (CTSI), a partnership among eight academic and clinical organizations. Our efficiency team included a process engineering expert, a physician-scientist, a quantitative analyst, and an experienced CTSA evaluator.

## Methods and Results

First, in order to inform our research, we searched PubMed, ProQuest Central, and Google with a variety of keywords, including LSS, barriers, translational research, and continuous improvement. We reviewed over 30 papers. Next, within a LSS framework, we organized our efforts using the Six Sigma define, measure, analyze, improve, and control (DMAIC) approach [15] and used a variety of tools drawn from both academic and LSS traditions. The present report focuses on the first three phases of DMAIC — defining, measuring, and analyzing the problem, but we also discuss the use of LSS tools to initiate the Improve phase, when we seek to mitigate specific research barriers.

### Define and measure

Based on the prior literature (e.g., [1]), we defined our problem as: *Researchers working in CTSI institutions experience many barriers that seriously hamper their ability to make medical discoveries and translate them to the bedside and community.* Further, they do not have an organized approach to identify and prioritize them.

In our Measure phase, in order to inform our LSS barrier identification efforts, as previously mentioned, we first identified barriers through a literature review, including work by the CTSA hub located at the University of Florida. In addition to a published qualitative analysis,[16] the University of Florida CTSA investigators shared summary results from a series of surveys conducted from 2011 to 2015. These annual satisfaction surveys included a section on perceived barriers to research. Further, to acquire a deeper understanding of our local barriers, we conducted a series of semi-structured interviews with CTSI stakeholders in the research process [17]. Stakeholders included individuals managing research relevant processes (e.g., institutional review board administrators, department business managers) and a mix of junior, mid-, and senior-level researchers at institutions affiliated with our CTSI. Twenty-three interviews were conducted over an 11-month period, using an interview guide (Appendix 1 in the Supplementary Material) aimed at: (1) eliciting barriers to research that the stakeholder had recognized, (2) understanding their knowledge of the services offered by CTSI, and (3) identifying services or support they believe would better support their research and research at CTSI institutions more broadly. The confidential face-to-face interviews were conducted by members of the quality and efficiency (Q&E) team, who summarized the interview, then returned the summary to the interviewee to obtain clarifications, corrections, and additional insights post-interview. These semi-structured interviews exposed multiple barriers we had not previously recognized; these were subsequently added to a graphical LSS tool that we describe next.

**Fig. 1.**
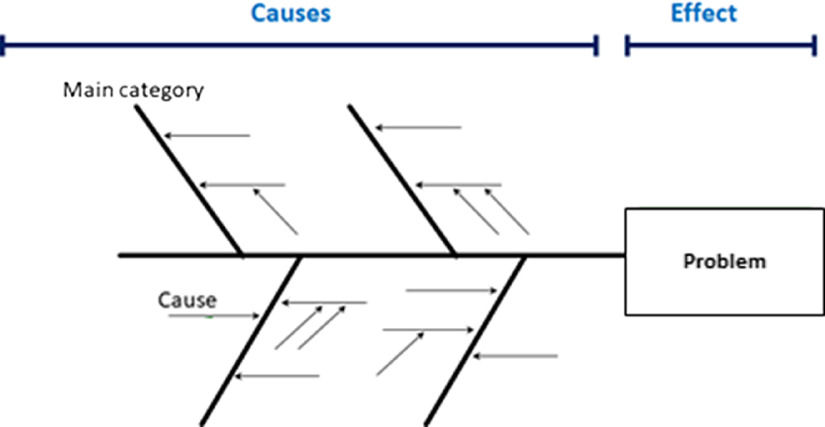
Cause-and-effect (fishbone) diagram approach.

Once the initial list of barriers was compiled, we organized and displayed them using a graphical LSS tool: The cause-and-effect diagram [15]. This process engineering tool is also known as a “Fishbone Diagram,” since it displays the problem (research inefficiency) as the fish head and the causes (in this case, barriers to efficient research) as bones (Fig. 1). The tool presents causes (in our case, barriers) in a hierarchical format.

**Fig. 2.**
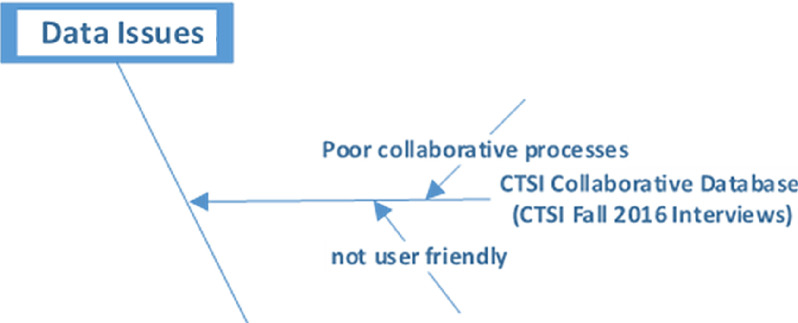
A portion of fishbone diagram.

For example, referring to Fig. 2, the top-level barrier is described as data issues. The lower level barrier (child), which is a type of data issue, is represented by a horizontal bone and is described as CTSI collaborative database. The various barriers (grandchildren) connected to the collaborative database appear as angular bones.

The full list of 27 barriers from the fishbone diagram is displayed in Appendix 2 in the Supplementary Material. This use of the fishbone diagram simplified presentations to key stakeholders (e.g., CTSI leaders and advisory board members), allowing them to see the scope of the problem and its hierarchical nature. It also provided the CTSI team with a graphical method to present projects aimed at mitigating specific barriers. In our hands, the fishbone diagram has been a “living” document; as new barriers are identified, they are categorized and displayed on the drawing and the filename version is incremented. As far as we are aware, this is the first published use of this tool to visualize research barriers.

### Analyze

Our next step was to identify the more important barriers. To do this, we added barrier questions to an extant annual survey (CTSI user satisfaction survey), which is used to inform CTSI leadership decision-making. We asked respondents to rank the importance of the aforementioned 27 barriers using a 10-point ordinal scale ranging from 0 (not a barrier) to 10 (extreme barrier — top 1 or 2 barriers to my research). To improve the user experience and increase the accuracy of response, a slider was used (Appendix 3 in the Supplementary Material). During 2019, this survey was sent to 417 individuals who are affiliated with CTSI service, support, and programs, 75 (18%) of whom responded., Of the respondents, 56% were male, 59% were engaged in clinical/patient-oriented research, 16% in basic/lab research, and 8% worked on social science research. We categorized research experience as 7 or more years (60% of respondents), 3–7 years (28%), or less than 3 years (12%). To ensure responses were informed, we limited the barriers portion of the survey to individuals with 3 or more years of research experience and who had conducted research within the previous year.

We present the top 10 barriers in Table 1 as measured by the mean score for each barrier. *Lack of Time to Conduct Research* was considered the most important barrier by the respondents.

**Table 1.** Barriers survey results

Barrier	Mean	StdDev
Lack of time to conduct research.	5.9	3.1
Inadequate/insufficient human subjects (IRB) process.	5.4	3.2
Insufficient expertise to support the use of “big data.”	4.9	2.9
Difficulties finding available resources and services to support research.	4.9	3.0
Unawareness of shared resources and equipment.	4.7	3.3
Lack of availability of database management experts and support.	4.3	3.1
Lack of availability of research coordinator support.	4.1	3.5
Difficulties in accessing participants for research.	4.0	3.3
Barriers between CTSI partners (MU, UWM, MSOE, etc.).	4.0	3.3
Lack of availability of biostatistical support and services.	3.7	3.1

**Fig. 3.**
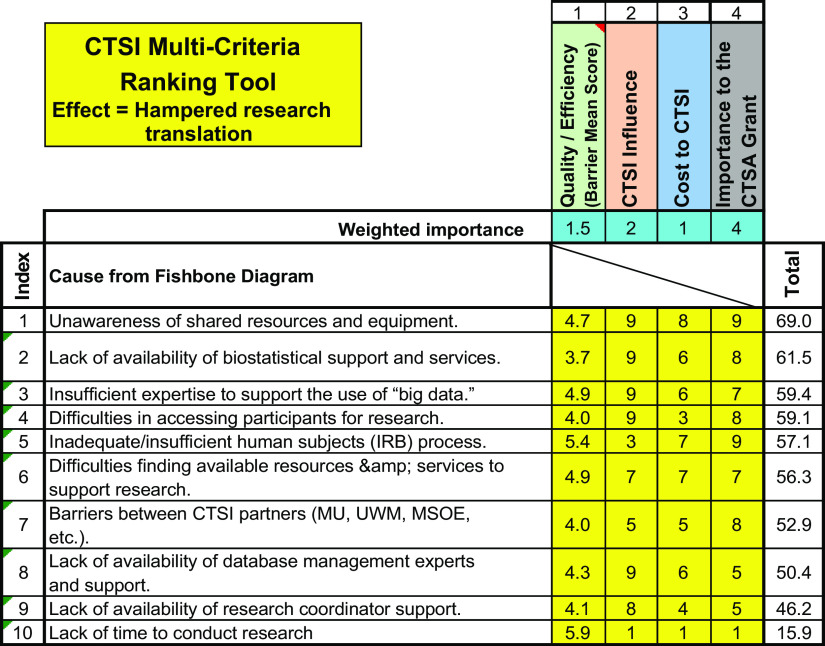
Multicriteria ranking tool and guidelines.

We next used a multicriteria ranking tool (Fig. 3) to incorporate pragmatic factors into our decision-making process This tool used four factors to assess which barriers were the most appropriate targets for CTSI efforts. First, Q&E, assessed the barrier’s impact on the overall efficacy and speed of the research process, as measured by the mean score from the barriers survey (for the remaining factors, project team members assigned a score from 1 to 10 for each barrier). The second factor, CTSI influence, reflects the perceived ability of the CTSI to influence the barrier, either directly or through relationships. The more influence CTSI has a barrier, the higher the score for this factor. Third, cost to CTSI, assesses the likely cost to CTSI of mitigating the barrier; the lower the cost to mitigate the barrier, the higher its score on this factor. Finally, importance to the CTSA grant reflects whether addressing the impediment had been an implicit or explicit “deliverable” in our funded proposal. If it was judged to be an explicit deliverable, it was ranked highly.

Next, in consultation with the local CTSA management team, we weighted the importance of each of the four factors to decision makers. Not surprisingly, given our dependence on grant funding, importance to the CTSA grant was weighted highest (weight = 4), while cost to the CTSI was weighted lowest (weight = 1).

Comparing the multicriteria rank of “*Lack of Time to Conduct Research”* and “*Unawareness of Shared Resources and Equipment*” illustrates the importance of this step. Although “*Lack of Time to Conduct Research”* was identified as the most important barrier to research in our CTSI user survey, our multicriteria ranking tool suggested that it was nowhere near the best target for intervention. It scored low on CTSI as the CTSI does not have an influence on the time faculty members have protected for research – and “Importance to the CTSA Grant” increases in faculty protected time was not a key NIH expectation in either current or expected requests for CTSA awardees. Instead, our tool scored *Unawareness of Shared Resources and Equipment* as the best target for intervention. To illustrate how the calculation was executed, we note that the impact of *Unawareness of Shared Resources and Equipment* on Q&E was ranked as moderate (4.7). However, given that the CTSI is able to directly address this barrier, it was ranked as a high impact (9). Likewise, since the projected cost to address awareness is low and awareness of CTSA support capabilities is a grant imperative, both were ranked as having a high impact. Thus, the multicriteria ranking tool score is (4.7 * 1.5) + (9 * 2) + (8 * 1) +(9 * 4) = 69.0. In contrast, note that *Lack of Time to Conduct Research* receives a score of only (5.9 * 1.5) + (1 * 2) + (1 * 1) + (1 * 4) = 15.9.

Thus, our multicriteria ranking tool is complementary to the information we gain from our survey of CTSI researchers, described above. The tool allows us to incorporate input from subject-matter experts and important stakeholders to calculate multicriteria rankings of the expected value of focusing CTSI efforts on mitigating specific barriers, enabling rationale selection of intervention targets. By using a formal, defined, process, we are more able to explain to leaders why a specific barrier is (or is not) an appropriate target for CTSI resources.

### Improve Phase

**Fig. 4.**
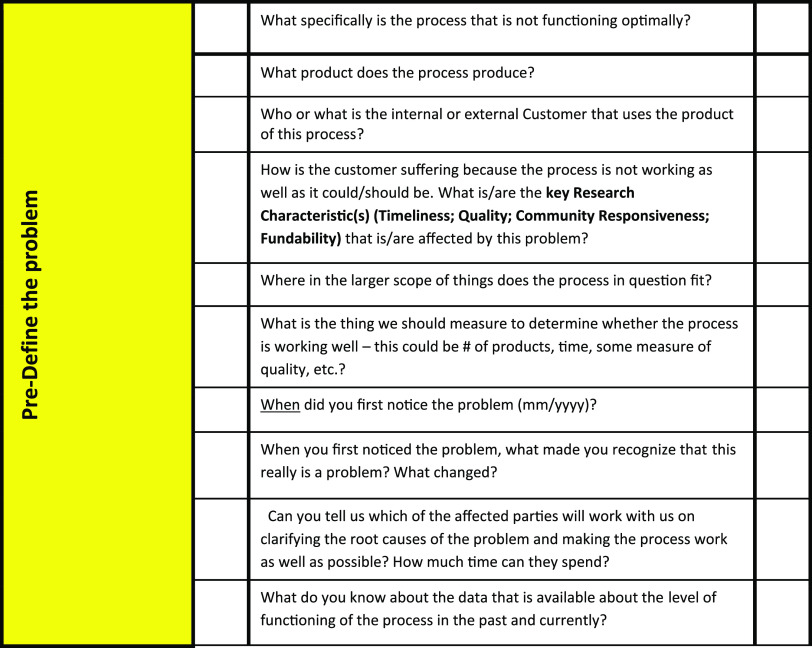
Scoping document.

While occasionally a barrier might be easily mitigated, in our experience, most of the barriers that have come to our attention are of sufficient complexity that we have used the DMAIC approach for projects to address them. To facilitate the define (problem definition) step in the process of addressing a barrier, we use a modified Six Sigma project definition worksheet (Fig. 4). Working alone, or with a project team member, the project sponsor completes the worksheet to begin the project scoping process; the worksheet then guides our team’s initial conversations with the project sponsors and provide an initial point of engagement with self-guided project teams. This is similar to the method described by others [11]. An example of a resulting project was one which helped to address the barrier “Unawareness of shared resources and equipment”. The project was conducted with one of the CTSA collaborating institutions such that a team of industrial engineering students worked with the Medical College of Wisconsin Department of Medicine. The DMAIC approach was used by the students, the current process was mapped, and a new improved process was successfully developed and documented along with an equipment tracking tool.

## Discussion

We have presented a novel stepwise approach to the problem of identifying and addressing barriers to efficiently conducting high-quality translational research, based upon the DMAIC problem-solving process. This approach allowed us to objectively determine a comprehensive set of these barriers and to funnel down to key targets. Furthermore, our approach’s novel use of hierarchical visualization (i.e., fishbone diagram) and a multicriteria ranking tool, provided effective means of communication with CTSI leadership.

While these techniques have been widely used in various industries, we believe they have not been widely used by CTSA awardees. We believe that future users in CTSA environments should describe their implementation of these and similar techniques, since their usefulness may vary across institutions. We also encourage descriptions of successful resolution of specific problems [13, 14]. Broader reporting of positive and negative experiences at individual CTSA awardees may reveal factors that influence whether specific solutions work at some institutions but not others.

This project has implications for the increasing emphasis on dissemination and implementation (D&I) activities and D&I science among CTSA hubs. Originally conceived as a method to enhance the adoption and integration of medical innovations into everyday clinical practice [18, 19], several CTSA hubs have recognized that similar processes can enhance the translational research process. Instructive examples [20, 21] and a theoretical model for integrating D&I into a translational research framework have been published [20]. Since these studies have demonstrated that D&I efforts can enhance the impact of a wide range of CTSA activities, the techniques we describe can help CTSA leaders prioritize areas where D&I resources, which are often in short supply, are best allocated. Moreover, the use of the prioritization process we suggest, which allows for comparison of disparate areas of research, may suggest that D&I requires increased attention by hub leaders.

We do note that while several authors have emphasized D&I as a method to enhance translation across the T1–T5 spectrum, we have focused on increasing the efficiency of research in general – most, but far from all, of the barriers we discussed are significant within a translational stage, though some (e.g., barriers to collaborating across partners) clearly have implications for translation across stages. When one hub identifies a method to increase efficiency, D&I has a crucial role in making sure that that method moves rapidly within the CTSA consortium and beyond. Indeed, given that the value of the processes we describe herein has been recognized for decades in other industries, D&I may have an important role to play in ensuring that they are adopted across the CTSA consortium.

We note several caveats regarding our description and identification of the barriers. First, we identified barriers at just two CTSA hubs; other CTSA hubs may have other barriers we did not identify. Within our hubs, both our surveys had limited response rates, so that non-response bias could be present. However, these response rates are not lower than other surveys of translational researchers [22–24]. Similarly, while we conducted just 23 semi-structured interviews at one of our hubs, we note that many reports in the literature have been based on fewer interviews [25–28]. In addition, we note that the barriers we identified are broadly similar to those described in other works.

We also note that while all CTSA quality improvement teams likely have access to experienced medical researchers, not all have access to robust process engineering expertise (the Milwaukee School of Engineering is a CTSI institution that does have such expertise). For example, it is not trivial to translate barriers from a fishbone diagram into a set of meaningful survey questions or develop a qualitative ranking tool that captures leadership priorities. At the same time, we realize that, in many cases, such expertise may indeed be present within an affiliated medical center’s quality improvement department [9–11].

Finally, we note that others may use different LSS tools or implement them differently. For example, one alternative to using the fishbone diagram for data visualization is the affinity diagram [29]. Similarly, others could question several choices we made for our qualitative ranking tool. For example, one could use a metric other than the mean score of responses as the ranking for a barrier’s impact on research Q&E or consider a larger range for ranking within the tool. Others could also use different weights or even different considerations to inform a *pragmatic* decision about what barriers should be targeted for intervention and score them differently. For instance, we note that more quantitative approach, such as the analytical hierarchy process (AHP) [30] may likewise be utilized. Further, it is noted that the weights and considerations themselves may be modified over time if the decision-making culture changes. We believe our approach, which reflected the input of multiple stakeholders, was useful, but welcome efforts to improve it.

In summary, we note that use of process engineering tools provides a structured approach that may assist CTSA hubs in identifying the processes where their efforts can most favorably impact the Q&E of translational research at their institution. These techniques also encourage hub leaders to explicitly consider the factors likely to influence successful intervention on these processes. Lastly, the use of explicit techniques such as the one we outline facilitates clear communication about why specific interventions are undertaken by CTSA hubs.

## Supplementary material

For supplementary material accompanying this paper visit https://doi.org/10.1017/cts.2020.522. click here to view supplementary material
