# Supplementary material for: Using a process improvement approach to identifying barriers to research in a CTSA hub environment
Source: J Clin Transl Sci. 2020 Aug 19;5(1):e30. doi: 10.1017/cts.2020.522 (PMC8057493; doi:10.1017/cts.2020.522)
Supplement: Supplementary file 1 [file S2059866120005221sup001.docx]

**Appendix 1, Interview guide**

**Appendix 2: Barriers found on CTSI Fishbone Diagram**

**Appendix 3, Portion of Survey Instrument**
